# Mechanical Ventricular Assistance as Destination Therapy for End-Stage Heart Failure: Has it Become a First Line Therapy?

**DOI:** 10.3389/fsurg.2015.00035

**Published:** 2015-08-03

**Authors:** Massimo Bonacchi, Guy Harmelin, Marco Bugetti, Guido Sani

**Affiliations:** ^1^Cardiac Surgery, Department of Experimental and Clinical Medicine, University of Florence, Florence, Italy

**Keywords:** end-stage heart failure, ventricular assist devices, heart transplantation, destination therapy, quality of life

## Abstract

Patients with end-stage heart failure have poor quality of life and prognosis. Therapeutic options are scarce and are not available for all. Only few patients can be transplanted every year. Several medical and surgical strategies have shown limited ability to influence prognosis and quality of life. In the past years, technological progress has realized devices capable of providing appropriate hemodynamic stabilization and recovery of secondary organ failure. Recently, these devices have been assessed as definitive treatment for patients who do not qualify for transplantation or/and instead to transplantation (“destination therapy”). This indication is increasingly considered following the results of newest clinical study reporting long-term survival without device correlated adverse events using last generation devices, and acceptable quality of life. The current knowledge about destination therapy and some original data from the DAVID Study (an Italian multicenter prospective study designed to evaluate the patient’s survival rate and quality of life of patients implanted with these new devices as long-term support or destination therapy) are summarized herein.

**Key Points:**

– End-stage heart failure (NYHA class IV or stage D) is a vastly growing problem, with a poor prognosis and limited therapeutic options.
– Heart Transplantation is nowadays the “gold standard” treatment albeit its inability to serve to the current demand let alone the future one.
– The REMATCH study has demonstrated that first generation implantable devices, even with their high number of complications (infectious and thromboembolic events), allowed an improvement of quality and duration of life compared to medical therapy. Further studies have shown that the evolution of VAD technology has reduced device-related complications and consequently improved survival and quality of life of patients with results, according to some authors, similar to cardiac transplantation at least at 2 years from the implantation.
– Biotechnology advancements have led to the creation of a new generation of implantable mechanical assist devices: the continuous-flow rotary pumps. Recently entered into clinical use, they seem to represent a promising solution to end -stage
– heart failure allowing long-term assistance and an adequate standard of living (Destination Therapy/Bridge to Life).
– The DAVID Study, an Italian multicenter prospective study, reports encouraging results in terms of late outcome and quality of life in patients implanted with newest continuous-flow devices as destination therapy. More and more numerous studies are needed to confirm our initial data and for laying the foundations for this new therapeutic frontier face to cardiac transplantation.

## Introduction

Advanced heart failure is an epidemic contributing considerably to the overall cost of health care in developed nations. The number of people afflicted with this complex syndrome is increasing at an alarming pace with this trend will likely continue for many years.

End-stage heart failure may present as an acute event or as the terminal stage of a chronic heart disease. In its acute refractory form, initiating mechanical support relies heavily on the assessment of the recovery potential of cardiac function and the patient’s possibility to be a candidate for transplantation or definitive mechanical assistance.

In the case of patients with terminal chronic heart failure, the therapeutic procedure is more complex. We are faced with patients who have poor prognosis and poor quality of life as encumbered by the presence of symptoms even at rest, require frequent hospitalizations and have complex, difficult to manage drug therapies. In this cohort of patients, the quality of life is very poor and mortality rate at 1 year that is approximately 50% (1, 2). Key treatment is heart transplant: with more than 85% 1-year survival and approximately 70–75% survival at 5 years, according to the registry of the International Society for Heart and Lung Transplantation (3). It is now clear that transplantation alone cannot meet all its demand and the availability of hearts for transplantation will always be a limited resource. Furthermore, even after optimizing the rate of donations per million inhabitants with the increase of older donors will not increase the actual availability of transplantable hearts (4). Moreover, the transplanted hearts are not immune to complications, with implications in survival and quality of life: this is evident from the data register collected by the International Society of Heart and Lung Transplantation (ISLHT) (see Tables 1 and 2) (3).

**Table 1:** **Prevalence of complications at 5 and 10 years in patients with heart transplant (modified by 2014 ISHLT Registry)**.

Complications	After 5 years (%)	After 10 years (%)
Hypertension	92	…
Renal failure	52	68
Abnormal creatinine <2.5 mg/dl	33	39
Creatinine >2.5 mg/dl	15	20
Dialysis	3	6
Renal transplant	1.1	3.6
Dyslipidemia	88	…
Diabetes	38	…
Cardiac allograft vasculopathy	30	50

**Table 2:** **Prevalence of malignant tumors at 1, 5, and 10 years after heart transplantation (modified by 2014 ISHLT Registry)**.

Tumors	At 1 year (%)	At 5 years (%)	At 10 years (%)
Absence of tumors	97.4	85.8	72.3
Tumors (all types)	2.6	14.2	27.7
Type of tumors			
Skin	1.3	9.4	19.6
Lymphatic	0.5	1.1	1.7
Other	0.6	4.1	8.7
Unspecified	0.2	0.3	0.3

Other therapies, both medical and surgical, show limited ability to affect the prognosis and quality of life (1, 4, 5).

The quite recent introduction of next generation left ventricular assist devices (VAD), which guarantee operational reliability for long periods, small footprint, and excellent quality of life, has changed the treatment possibilities for these patients (6–8). Based on latest clinical experience, we can now implant these new “artificial ventricles” in patients who have contraindications to transplantation. The future perspective is to realize a “Destination Therapy” and a “Bridge to Life” in these patients with a survival potential and quality of life similar if not better than the transplantation.

The initial data coming out of the DAVID study, a multicenter prospective clinical trial, as other worldwide similar studies data, designed to evaluate the survival and quality of life in patients with end-stage failure VAD-supported seem to support this encouraging perspective.

## Development of Ventricular Assist Devices: From Extracorporeal Pulsatile Pneumatic Pumps to Intra-Ventricular Continuous-Flow Rotational Pumps

The possibility of replacing human organs with artificial organs has stimulated medical research since the beginning of the twentieth century. In the 60s, with the advent of the heart–lung machine and the increased number of complex cardiac interventions, came a boost in development of systems for temporary ventricular assistance: In 1963, DeBakey implanted the intra-thoracic first pump in a 42-year-old man who had underwent an aortic valve replacement complicated by post-cardiotomy syndrome. For many years, the improvements were only partial and, in fact, both short- and long-term devices were implanted only in a few dedicated centers, which even in highly selected patients were unable to obtain satisfactory results. The major problems encountered were linked to specific factors including inadequate biocompatibility (see Table 3). As technology advanced many of these issues have been resolved and various new devices have been redesigned and marketed for clinical use.

**Table 3:** **Issues with cardio-circulatory assist devices**.

The energy source, consisting of very large and heavy compressors
Bulky and short life batteries
The thrombogenicity of the contact surface with circulating blood
The size of the device, too big to consider for long-term intra-thoracic implantation
The need for extensive connectivity measures with the exterior in order to connect the device to the energy source and to the controller
The high rate of bleeding complications and infectious diseases

The initial concept with pulsatile-flow pumps was to construct a device that would mimic blood flow as close as possible to the human physiology. The blood volume was moved by means of a pneumatic system driven by a compressor. The first clinical application was implemented with *extracorporeal systems*, in which the pump and the source of energy were placed outside of the patient body. The ventricles were connected by means of cannulae and could replace the left ventricular function, right or both depending on clinical needs. Assistance could last for a relatively short period (up to a few weeks). It was burdened by a high rate of complications and required the patient to stay in his bed at all times. The patients could only be partially mobilized and they remained hospitalized until transplantation.

Second to come were the *para-corporeal systems*. Here, the power source was external and the pump was fixed to the patient’s external surface, which could, thanks to the development of smaller compressors, get out of bed and walk around. These systems can provide both mono- and bi-ventricular support and can be used both as a “bridge to transplantation” and as a “bridge to recovery” and are still used to date.

**Figure 1:**
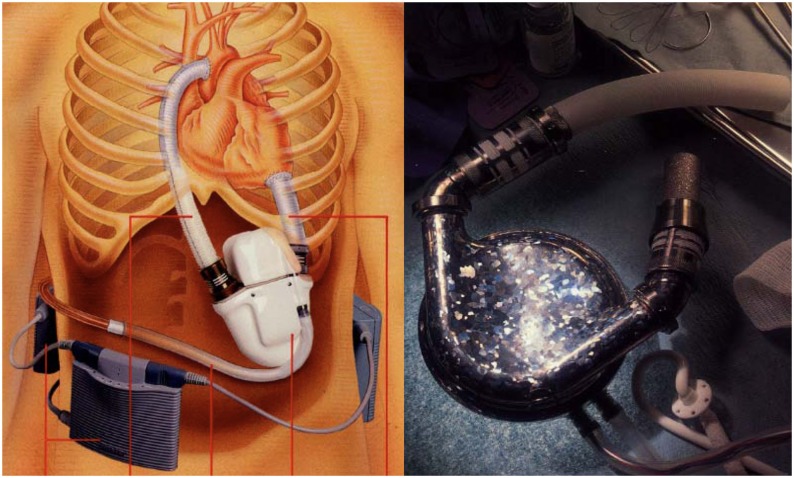
**Fully implantable pulsatile ventricular assist devices: to the left Novacor (Source: World Heart, www.worldheart.com, accessed December 31, 2014.), to the right HeartMate I XVE (Source: Thoratec, www.Thoratec.com, accessed December 31, 2014.)**.

Further significant advancement was represented by *intra-corporeal systems*; totally implantable pulsatile-flow pumps, fed first via pneumatic systems, and subsequently electrically. These are devices are used exclusively for left ventricle assistance. The implant, due to its size is must be placed in the abdominal cavity and can be intra- or extra-peritoneal. The connection is made with the heart’s chambers via cannulae connected to the left ventricle and the aorta. A percutaneous cable is used to connect the device to an external power supply and control unit. The most advanced models used to date are Novacor and HeartMate I XVE (Figure 1). Major issues presented in this category are size, which make it difficult to implant, the presence of large external connection lines that may be due to cause frequent infections (30–50% of cases), and thromboembolic events (9, 10).

**Figure 2:**
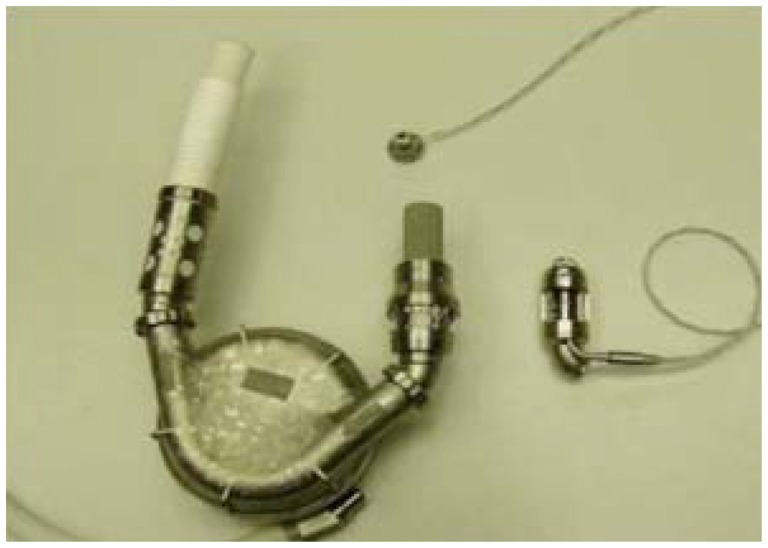
**Size comparison between pulsatile-flow VAD (left) and continuous-flow VAD (to the right)**.

This group of devices presented for the first time the possibility for a true bridge to transplantation with thousands of cases, with one reaching more than 6 years of life (11). In contrast to the first generation of pulsatile pneumatic and after, electric pumps, substantial development has led to the construction of non-pulsatile, miniaturized rotational pumps. Electrically powered continuous-flow pumps are based on a rotating system that creates propulsive energy driving a continuous flow of blood. For many years, it was thought that this type of flow is not tolerable for long periods in mammals. This *presumption* has been rebutted by all preclinical and clinical studies carried out (12, 13). Several types of pumps exist, such as extracorporeal and para-corporeal systems commonly called “centrifugal pumps” and used primarily as a circulatory support systems in the short/medium term, i.e., as a bridge to definitive treatment (Jostra Rotaflow, TandemHeart, Levitronics Centrimag). Continuous-flow pumps of rotary type with or without mechanical suspension (in this case generally centrifuge) were the first to be proven effective in clinical use and particularly suitable for assistances of medium and long duration (destination therapy). These devices are of limited size with a rotor wing that rotates at variable speeds (5,000/12,000 rpm) and generates a flow rate of up to 10/12 l/min., having significant advantages (14–17) (see Table 4). The main advantage is the use of minimally invasive implantation techniques (mini-access, beating heart and, eventually, without CPB) with considerable reduction of surgical trauma (Figure 2). As regards the long-term impact, it seems that the continuous flow is well tolerated. Initially, for a few weeks, patients may experience changes in baroreceptor activity, in the release of catecholamines, the lymphatic pumps, renal cortex flow, and vascular permeability. Potentially contributing to a certain degree of fluid retention and edema that usually resolves itself in a short time. It has also been shown that there is a reduction of the oxygen consumption of 20% and an increase in the coronary flow (12).

**Table 4:** **Main differences between ventricular assist devices (VAD) with pulsatile flow and VADs with continuous flow**.

**Advantages of continuous-flow VADs versus pulsatile-flow VADs**
Smaller dimensions (better compliance and easier implantation)
Simple structure with fewer moving parts (less risk of mechanical failure)
No filling chamber (less chance of stasis and thromboembolic events)
Reduced energy consumption (smaller batteries and greater autonomy)
**Hypothetic disadvantages (with negative clinical implications – unproven)**
A certain degree of hemolysis, usually well tolerated by patients
The long-term effects of systemic non-pulsatile flow are still not known
Control mechanisms (feedback) of the speed of the pump, and therefore, the flow generated are complex and not yet optimized

**Figure 3:**
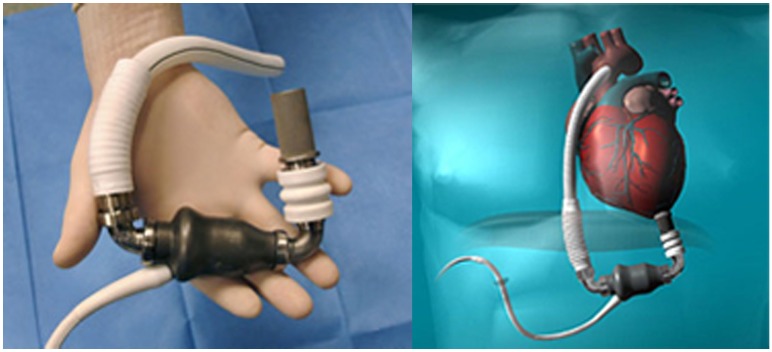
**HeartMate II (Source: Thoratec, www.Thoratec.com, accessed December 31, 2014.)**.

Among them, the first to receive FDA approval for Destination therapy in 2010 was *HeartMate II LVAS* (in 2008 was approved for “bridge to transplantation”) (Figure 3).

**Figure 4:**
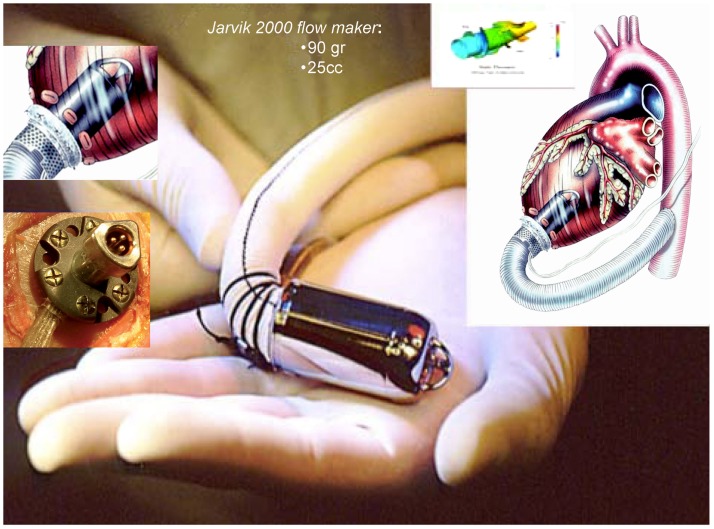
**Jarvik 2000 Flowmaker**. In the boxes, specific aspects of the device: intra-ventricular system, retro-auricular fitting (Source: JarvikHeart, www.jarvikheart.com, accessed December 31, 2014.).

The *Jarvik 2000 Flowmaker*, is the smallest VAD, to date, applicable for total ventricular assistance as destination therapy (5.5 cm in length and 90 g of weight); it is for many aspects, a peculiar system within the axial devices group, with advantages that make it today one of the most safe and reliable (Figure 4). The device is completely housed inside the left ventricle with a duct outflow anastomosed to the descending aorta making it extremely “integrated” both anatomically and functionally with the ventricle (7, 16, 17), thus, substantially reducing the space and surfaces in contact with blood.

First in Europe (2005) and recently in United States (2012), the FDA approved a trial to evaluate the Jarvik 2000^®^ for destination therapy indication called – Randomize Evaluation of Long-term Effectiveness intra-ventricular VAD (RELIEVE). It is the only approved system for clinical use that has its power supply system implanted behind the ear, on the left temporal bone. This technique (derived from cochlear implants) has shown considerable advantages in terms of resistance to infection and management by the patient, with consequent improvement in the quality of life for patients in which the indication is an alternative to heart transplantation.

**Figure 5:**
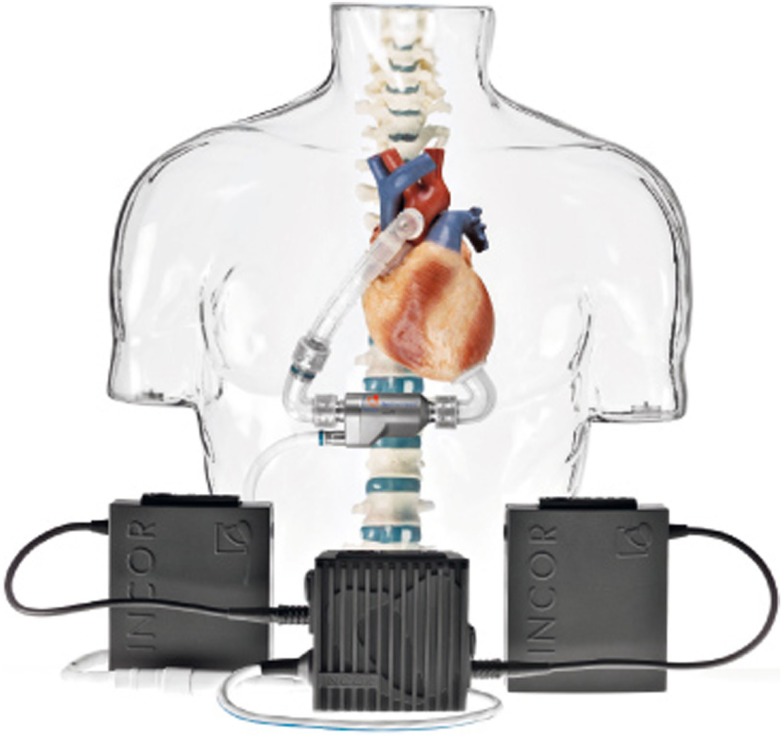
**Berlin Heart Incor (Source: Berlin Heart, www.berlinheart.com, accessed December 31, 2014.)**.

**Figure 6:**
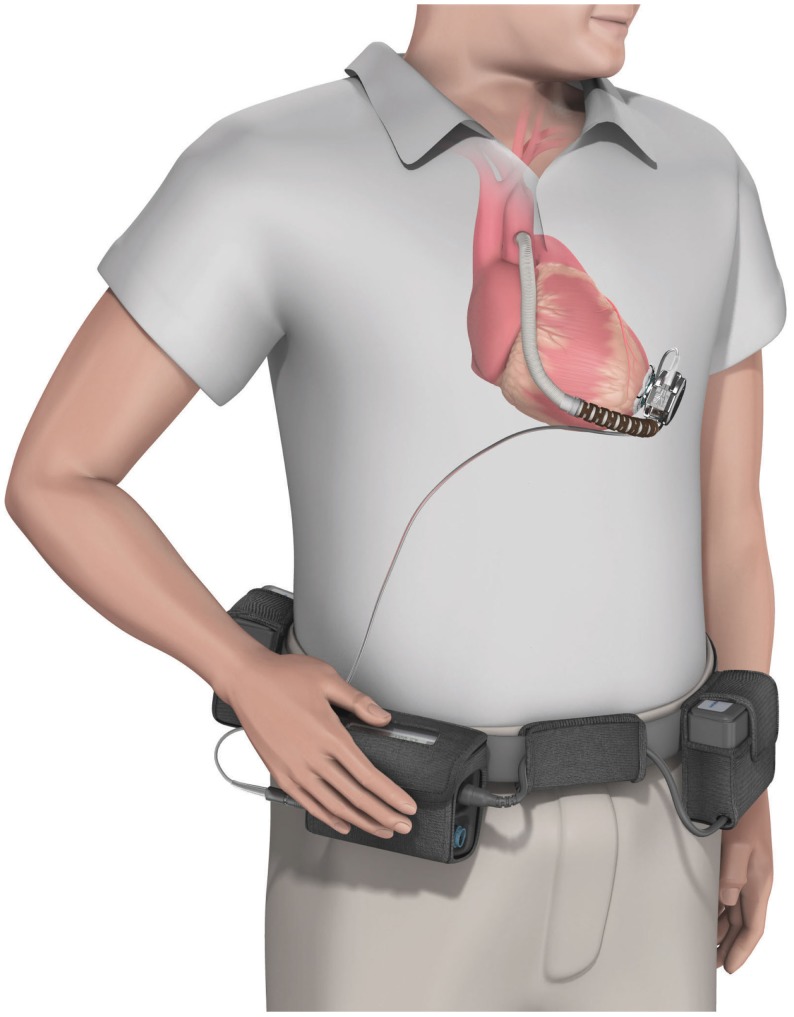
**The HVAD (HeartWare Corp.) ventricular assist device**. (Source: http://www.carmatsa.com/, accessed December 31, 2014.).

“Third generation support devices,” already in clinical use, are smaller rotational pumps, designed with a magnetic levitating rotor (similar to a propeller). These pumps are simple in maintenance and at the same time less harmful to blood cells, thereby reducing hemolysis. The blood flow is not axial (inflow and outflow axes are arranged in a 90° angle) and they run with lower rotation speed of 1000–2500/min. The moving part “impeller,” spins blood to generate up to 10 l/min of blood flow. The pump is connected to the controller via a thin driveline, which is tunneled just the upper right quadrant of the abdomen. Two examples of third generation devices that are clinically employed are as follows:

- *Berlin Heart Incor pump*: this device uses magnetic suspension technology preventing any contact between the rotor and fixed components. The pump weighs 200 g and has a diameter of 3 cm and a length of 12 cm. It generates a flow of blood up to 14 l/min. The connecting lines to the battery and control unit pass through the abdominal wall with the inner surface of the device coated with heparin (Figure 5);
- *HVAD* (HeartWare Corp.): with a displacement volume of 45 ml, and weight 145 g this device has a flow capacity of up to 10 l/min. The HVAD uses a wide-blade impeller design to maximize performance and hemocompatibility, size minimization, long-term reliability, and overall system efficiency. The impeller is suspended in place by combination of passive magnetic and hydrodynamic bearing systems to avoid mechanical contact and wear. The impeller suspension system uses a passive magnetic bearing for radial stiffness. The axial alignment of the center-post magnet stack is set to provide an axial force that pushes the impeller toward the forward housing (the assembly with the inflow cannula). Physical contact between the housing and the impeller is prevented by a thin blood-film generated by the hydrodynamic bearings. The hydrodynamic bearings feature a shrouded design that is intended to maximize the blood-film thickness and improve surface washing (Figure 6).

## Total Artificial Heart

**Figure 7:**
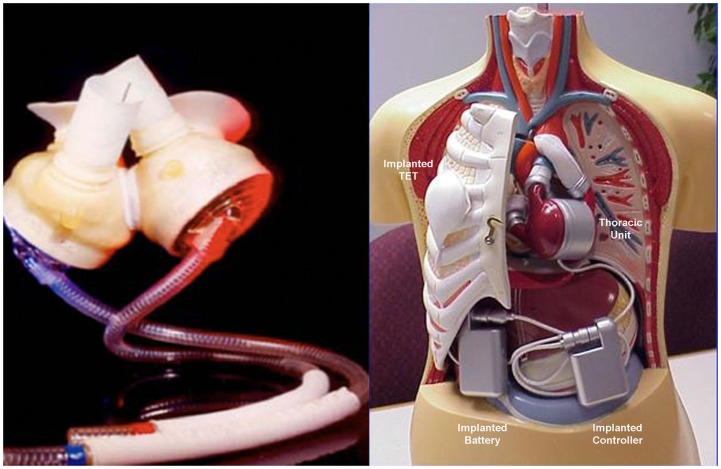
**Two devices approved by the FDA for the complete replacement of the heart, the Jarvik-7-CardioWest (left) (Source JarvikHeart, www.jarvikheart.com, accessed December 31, 2014) and the AbioCor (right) (Source: AbioCor, www.abiomed.com, accessed December 31, 2014)**.

The total artificial heart (TAH) is the most advanced implantable pump system. As first VAD devices, it is mostly used as a “bridge to transplant.” It is necessary to ensure a balance between stroke volumes of both ventricles and adjust the flow generated to the physiological needs. Among the TAH s available, two are in an advanced stage of experimentation and approved by the FDA: *The Abiomed AbioCor Total Artificial Heart* and the *Jarvik 7-CardioWest* (Figure 7). At the moment, their clinical use is limited, albeit an important development is expected the near future. The indications are reserved for patients waiting for a heart transplant for which any other type of assistance are excluded. The most experienced cardiac surgery center with TAH implantation is Bad-Oeynhausen in Germany, with more than 160 CardioWest devices implanted and an operative mortality at approximately 67% due to the extremely severe conditions of patients (18).

**Figure 8:**
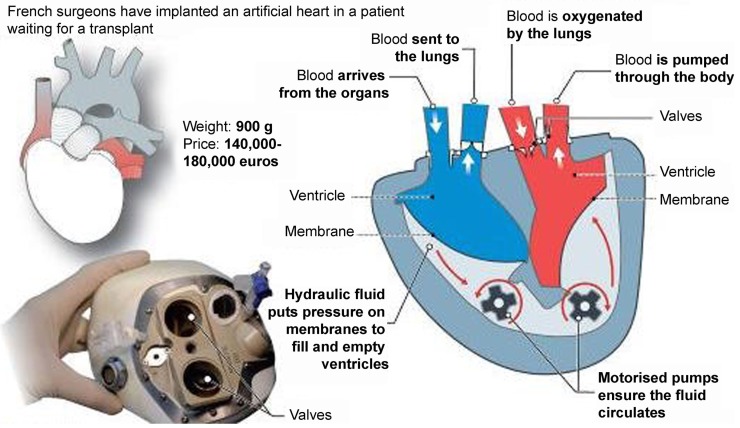
**The CARMAT (Carmat, Velizy, France) total artificial heart (Source: CARMAT, www.carmat.com, accessed December 31, 2014)**.

Recently, a new TAH, the *CARMAT (Carmat, Velizy, France)* (Figure 8), was introduced for clinical use with the ambition to became the first TAH used for “Destination therapy.” The first CARMAT was implanted in December 2013 (the patient died after 75 days due to a device failure), the second implanted in August 2014 with the patient currently home. The device contains two ventricles, each with a blood compartment and a driving fluid compartment, separated by a flexible hybrid membrane (in polyurethane). All the blood-contacting surfaces are covered with bovine pericardial tissue with the use of bio-prosthetic valves at the inlet and outlet of each blood compartment. This might permit a reduction of anti-coagulation therapy. Electro-hydraulic pumps create a systolic and a diastolic phase by moving the silicone fluid and deploying the membrane. The stroke volume and the beat rate of the prosthesis adapt automatically in response to changes in preload, detected by the pressure sensors located in the device. The resulting pulsatile blood flow ranges from 2 to 9 l/min with a flow adjustment on the right side to correct for the bronchial shunt. A percutaneous driveline delivers power to the prosthesis and allows exchange of data and inputs. This implantable prosthesis is still quite large, it weighs 900 g; studies performed by the manufacturer claimed that it fits 65% of patients (86% of which were men) (19, 20).

## Clinical Use of Cardio-Circulatory Assist Devices: Indications and Strategies for Implantation

It is true that the criteria for implantation of the cardiovascular assist systems are not fully defined because there are not enough established clinical trials (21–23). There is, however, strong evidence that may serve as guidelines. Mechanical assistance is used mainly in cases of post-cardiotomic shock or other acute situations in which we consider the possible recovery of cardiac function (bridge to diagnosis, bridge-to-bridge, or bridge to recovery) (24–26) or in terminal patients with conditions that do not allow transplant due to critical state, in order for them to recover from shock and multi-organ failure, making them candidates for transplantation (bridge to transplantation or bridge to candidacy) (26–28). In such cases, it may be necessary to support a single ventricle or bi-ventricular. The results depend on the complications related to mechanical assistance and medical condition of the patient at implantation.

The encouraging results obtained with pulsatile electric VAD (5, 9, 21), further improved with the use of continuous-flow pumps (6, 13, 16, 17), allow us to take into account the use of VADs as definitive therapy or “destination therapy” in order to obtain “Return to Normal Life” or “Bridge to Life.” There is also the possibility, at the moment demonstrated only in a selected category of patients, to obtain some sort of “reverse remodeling” favored by decompression and functional rest of the cardiac cavities (23–26, 29–31).

In summary, the following potential goals of mechanical circulatory support can be defined according to the intention and the clinical situation:

1. Bridge to decision or bridge-to-bridge;
2. Bridge to recovery;
3. Bridge to candidacy;
4. Bridge to transplant;
5. Destination therapy.

## Destination Therapy: Current Perception and Clinical Implications of Cardiovascular Mechanical Assistance

The best estimates for the number of patients under the age of 65 years with chronic heart failure in the pre-terminal assess around 250,000 in the U.S. and 10,000 in Italy. Only 2000/2300 in the USA and 200/250 in Italy heart transplants are performed each year (1, 4, 7, 8). The difference, in theory, should be covered by mechanical devices. These numbers will increase several fold when we will start considering as candidates patients in NYHA class 3b and move the age limit to 75 years (4).

Rarely will we evaluate the possibility of a VAD implantation as a viable alternative to transplantation, often for economic reasons since its relatively initial high cost. Thus, without considering the savings in terms of hospitalization and therapy with transplanted patients. Furthermore, there is lack of confidence in the presented results in the literature as there are no randomized trials that compare the different devices available. There are, however, observational studies that are of high significance.

The “Randomized Evaluation of Mechanical Assistance for the Treatment of Congestive Heart Failure” (REMATCH) study (9) and “Investigation of Non-Transplant-Eligible Patients who are inotrope Dependent study” (INTREPID) (32) evaluated, in this regard, the two electrical, pulsatile, totally implantable VADs who had been most used clinically to that date: HeartMate I and Novacor.

The results of the REMATCH study (10) show that the survival of the VAD group compared to the medical therapy group, at 1 year are 52 versus 25% (*p* = 0.002), and at 2 years 23 versus 8% (*p* = 0.09). Moreover, there is a marked improvement in the quality of life in the VAD patients (Table 5).

**Table 5:** **Evaluation of quality of life in the two groups using rating scales of physical and mental wellbeing**.

Scale used	Evaluation at 1 year		*p*
	Patients evaluated/total (%)	Score	
Short form 36 questionnaire			
Physical state			0.01
LVAD group	23/24 (96)	46 ± 19	
Medial therapy group	6/11 (55)	21 ± 21	
Emotional state			0.001
LVAD group	23/24 (96)	64 ± 45	
Medial therapy group	6/11 (55)	17 ± 28	
Minnesota living with HF			0.11
LVAD group	23/24 (96)	41 ± 22	
Medial therapy group	6/11 (55)	58 ± 21	
Beck depression inventory			0.04
LVAD group	22/24 (92)	8 ± 7	
Medial therapy group	5/11 (45)	13 ± 7	
Classe NYHA media			<0.001
LVAD group	22/24 (100)	II	
Medial therapy group	7/11 (64)	IV	

*Modified by Rose et al. (9)*.

These results compared with those of transplanted patients do not seem exciting (3), it is necessary however to consider the severity of the patients conditions and their older age (average age is 65 years). Several of the deaths reported were related to dysfunctions of the device itself (35%), infections of the percutaneous abdominal the driveline (41%) and to further cerebrovascular events (10%) (Table 6). Similar results have been reported from the study INTREPID (33).

**Table 6:** **Causes of death in both groups**.

Cause of death	Group medical therapy	Group LVAD	Total
	Number of patients
Left ventricular dysfunction	50	1	51
Sepsis	1	17	18
Failure of VAD	0	7	7
Non-cardiovascular causes	0	5	5
Cerebrovascular events	0	4	4
Other cardiovascular causes	1	2	3
Pulmonary emboli	0	2	2
Acute myocardial infarct	1	0	1
Cardiac procedure	1	0	1
Operational bleeding	0	1	1
Unknown	0	2	2
Total	54	41	95

*Modified by Rose et al. (9)*.

A subsequent study (6) of 309 patients implanted between November 2002 and December 2005 and included in the FDA Destination Therapy Registry, divided the patients into two categories, high and low risk. Survival is 81 and 11% at 1 year and 48 and 0% at 2 years, demonstrating the need for a more careful patient selection. Another study (22) using the database of the REMATCH study divided its patients into two groups according to the period of implantation (1998–1999 and 2000–2001) and evaluated the outcomes and adverse events. This analysis deducted that a more careful selection and management of patients allowed better results; at 1 year 59 versus 44% and at 2 years of 38 versus 21% (*p* = 0.029).

## Clinical Implications of New Generation VADs: Devices with Continuous Axial Flow

The clinical introduction of VAD’s has allowed us to confirm preclinical data on the possible use of the continuous flow (12–14), and the important advantages in terms of efficiency, durability, thromboembolic complications, and infections (6, 7, 14, 16, 28). These advantages were confirmed by a study of 133 patients (NYHA class IV and UNOS status I and an average age of 50 years), which were implanted with axial pumps (HeartMate II). After 180 days, 100 patients (75%) achieved a major outcome of the study (cardiac transplantation, recovery of cardiac function or survival with mechanical support active). Furthermore, compared to the REMACTH study, there was a significant decline in complications: infections went from 3.49 to 0.37, stroke from 0.67 to 0.26; neurologic events from 0.67 to 0.26; and right ventricular failure from 0.30 to 0.08. This data are a prerequisite for the hypothesis for using VADs as an alternative to transplantation, especially of symptomatic patients (15, 19). The reliability of the system is demonstrated by the experience carried out in the year 2000 with a Jarvik device in a male patient of 60 years (16) who suffered from idiopathic dilated cardiomyopathy with severe terminal heart failure. Immediately after implantation, the patient regained an acceptable clinical condition and after about three months was in NYHA class I. He remained free of events related to the device for 7.5 years. His quality of life was excellent. The cause of death was not related to the device, as confirmed by the autopsy. It showed the perfect integrity of the pump and the absence of thrombotic appositions on it (7, 16).

Despite all efforts to optimize cardiac support devices some complications still occur throughout the period of support:

– *Bleeding*, particularly in the peri-operative period, but rarely disastrous with new devices (34)
– *Cerebral, Gastrointestinal bleeding, and peripheral thromboembolic complications* (35, 36), strictly correlated with the continuous flow that seem to promote gastrointestinal bleeding due to angiodysplasia or arteriovenous malformations and appears to be related to the different flow characteristics of these devices (37) and the anti-coagulation regimen administration (38). The pathophysiological mechanism seems to be related to the shear stress of continuous-flow devices that may cause proteolysis of the Von Willebrand factor. In addition, there is a prolonged activation of the fibrinolytic system, and some loss of platelets. In order to decrease the incidence of such events, screening for von Willebrand disease and gastrointestinal pathologies may be indicated before implantation of such LVAD systems.
– *Infections* are the most frequent complication in patients with a mechanical circulatory support system. Predisposing factors for local wound infection are age, diabetes, tension on the wound edges, and localized hematoma followed by bacterial colonization (39). Infection is often linked to the percutaneous driveline. The method of bone fixation seems to reduce drastically this nasty complication. One essential technical progress needed to optimize the destination option and simplify the daily life of such patients is the so-called “Transcutaneous Energy Transfer System” thus avoiding altogether the percutaneous energy driveline.
– *Right ventricular failure* and life-threatening arrhythmias (40). Playing a key point in the isolated left ventricular mechanical assistance: right ventricular failure is one of the most important causes of peri-operative and early postoperative mortality and morbidity following LVAD implantation: changes that occur after initiating left ventricular support should be followed very closely during early post-implant management in ICU. Echocardiography has clearly shown that after LVAD assistance started, the interventricular septum is pulled to the left and the RV free wall is distended. Before LVAD implantation, it is therefore very important to optimize RV function, but while pulmonary hypertension and the risk of severe RV failure are some of the major concerns during the assessment of candidates for “bridge to transplantation” these factors is only of modest importance in the setting of destination therapy. Implantation of a LVAD will typically lead to a significant decrease of pulmonary pressure and secondarily of right atrial pressure (28).
  Recently, an RV failure risk score (RVFRS) was proposed by Matthews et al. (41) composed of routinely collected, non-invasive pre-operative clinical data (vasopressor requirement; aspartate aminotransferase, bilirubin, and creatinine levels) effectively stratifies the risk of RV failure and death after LVAD implantation.
  Different diagnostic tools have been proposed to evaluate accurately pre-implantation right ventricular function. Recently (42), we applied RV deformation analysis by speckle tracking echocardiography for a deeper analysis of RV longitudinal function before and after LVAD implantation, founding important clinical implications for selection and management of LVAD patients.
– *Hemolysis* is most probably dependent on the pump design, whose shear forces affect the red blood cells when they pass through the pump (43).

## Mechanical Assistance as Definitive Therapy – Current Status

The improvement related to the use of latest generation VADs has led us to reconsider the clinical care path of patients with terminal heart failure. Assistance may be short, medium, or long term and should have as its aim the recovery of cardiac function (bridge to recovery) or transplantation (bridge to transplantation). Using an axial pump, patients can remain implanted for long periods (years) and time of a transplant can be decided upon with greater care.

Older patients (60–65 years) are typically placed lower on transplant lists. They are usually severely symptomatic, require frequent hospitalization, have a poor quality of life and a poor prognosis (1–2 years) (32). Therefore, they have few hopes to be transplanted and in case they would be, it will be with “marginal hearts.” Even in these hard cases, there is a clear indication of continuous-flow VAD implantation as definitive therapy or at least for the long-term (such as “destination therapy” and “bridge to life”). Similarly, patients who have an absolute contraindication to transplantation or relative (pulmonary hypertension, systemic diseases, cancer already made, etc.) have a well-defined indication for long-term VAD implantation (15, 28).

The financing of VAD as destination therapy and heart transplantation has to be considered in the global context of heart failure treatment. Essentially, various national reimbursement systems fear that with the establishment of alternative cardiac replacement procedures, a new considerable cost-push will be expected from the healthcare premium payers. However, the costs of LVAD procedures are lower compared to the costs generated not only by patients who undergo cardiac transplantation but also by patients with advanced heart failure that need complex multi-therapies, continue assistance, and several hospitalizations a year (22, 44).

## The DAVID Study and Conclusions

Given the recent technological developments and the results in terms of quality and duration of life that can be obtained with the latest generation of VADs, we wonder what will be their use in the near future and if they ever will be preferred over cardiac allografts. The rapid development in terms of miniaturization, reliability, biocompatibility, and flexibility, bring them closer to the characteristics of an “ideal VAD” (Table 7). New encouraging results have been obtained from clinical trials: the latest data from INTERMACS 2014 suggest that the survival rate at 2 years after implantation is analogous to that of the transplanted heart (45). The absence of large and well-designed prospective trials demonstrating unequivocally the effectiveness and efficiency of the new VADs makes it still difficult to convince cardiologists to direct their patients to VAD implantation and administrators to fund programs (22, 44). Several long-term studies are currently being pursued worldwide for different LVAD implanted for long-term or destination therapy (45, 46).

**Table 7:** **Characteristics of an ideal implantable ventricular assist device**.

Characteristics of an ideal implantable ventricular assist system
Biocompatible
Small
Absence of percutaneous lines
Reliable (10,000,000 beats/year or four billion rounds/year)
Low-energy consumption
Easy to implant and explant
Allowing quick discharge
Low cost

The Dispositivo di Assistenza Ventricolare ed Immissione a Domicilio (DAVID = Ventricular Assist Device and Back to Home) is an Italian multicenter prospective observational study intended to assess the survival, quality of life, and economic impact of axial pumps used as “Destination Therapy” and “Bridge to Life.” The mechanical assist device chosen was the Jarvik 2000 Flowmaker^®^. Due to its major characteristics (small size, “minimally invasive” implantation without extracorporeal circulation system and a retro-auricular driveline) it seemed to be particularly suitable for long-term assistance (Destination Therapy).

The study protocol include patients with end-stage heart failure who were not candidates for transplantation due to exceeding the age limit or the presence of specific medical conditions and/or hemodynamic. Contraindications for implantation of the VAD were moderate-to-severe right ventricular insufficiency, severe renal failure, or cancer with a poor prognosis of <2 years.

The study, which was led by our Center, was launched in June 2006. To date (December 2014), 143 consecutive patients were enrolled and underwent implantation of the Jarvik 2000^®^ Flowmaker. The operations took place in 18 Italian Cardiac Surgery Centers. Of the 143 patients, 9 patients were under the age of 18 years. As for adults, the average age was 62 years (29–75). The main demographic, clinical, and instrumental data are reported in Table 8.

**Table 8:** **Clinical and instrumental data of patients enrolled in a prospective, multicenter, observational study (DAVID)**.

**Demographic**	
Implantations overall (*n*)	143
Gender (M/F)	123/20
Adult patients (*n*)	134
Pediatric patients (*n*)	9
Average age adult patients (years)	62 [29–75]
Median age adult patients (years)	61
Average age pediatric patients (years)	15
BSA adult patients (m^2^)	1.9 [1.17–2.36]
BSA patients pediatrics (m^2^)	1.6 [1.4–1.68]
**Etiology**
Idiopathic dilated cardiomyopathy	53 (37%)
Post-ischemic cardiomyopathy	76 (53%)
Other^a^	14 (10%)
**Pre-implantation instrumental cardiac function evaluation**	
Cardiac index (l/min × m^2^)	1.9 ± 0.4
Systolic pulmonary artery pressure (mmHg)	52 ± 16
Pulmonary artery wedge pressure (mmHg)	23 ± 9
Pulmonary vascular resistance (WU)	4.3 ± 2.2
ECHO left ventricular end diastolic diameter (mm)	73 ± 12
ECHO ejection fraction (%)	21 ± 5
TAPSE – tricuspid annular plane systolic excursion (mm)	16 ± 4
INTERMACS level	3.0 ± 1.2

*Data are expressed as mean ± SD. Square bracketed are the maximum and minimum values*.

*^a^In particular, pediatric patients with five Duchenne muscular dystrophy cases, three hypertrophic cardiomyopathy, and one congenital muscular dystrophy*.

The implantation of the VAD was preferably performed with a minimally invasive procedure, using a left lateral thoracotomy without the use of extracorporeal circulation and with the driveline connected with a retro-auricular pedestal; in some cases, it was necessary to use extracorporeal circulation and a longitudinal median sternotomy due to hemodynamic instability and/or the need for associated procedures (tricuspid repair and/or mitral stenosis, aortic valve replacement, and ventricular repairs) (Table 9).

**Table 9:** **Surgical implantation technique**.

Surgical entry	Off cardiopulmonary bypass 81 (57%)	On cardiopulmonary bypass 62 (43%)
Left thoracotomy 110 (77%)	95 (87%)	15 (13%)
Sternotomy 33 (23%)	1 (3%)	32 (97%)

The assessment of the duration of the assistance to date (December 2014) is detailed in Table 10. Fifty-eight patients have been enjoying ventricular assistance for more than 1 year, 35 patients have been enjoying ventricular assistance for more than 2 years, 15 patients for over 3 years of support, 8 patients for over 4 years of support, and 1 has surpassed 5 years. The total duration of the assistance was >180 years/patient. During this period, no malfunction of the device was registered thus confirming the high reliability of the Jarvik 2000.

**Table 10:** **Duration of care with in patients enrolled in the DAVID study**.

Cumulative time on system (years)	183.6
Average time on system (days)	440
Average time on system of patients discharged out of hospital (days)	594

Of the 143 patients implanted, a total of 23 died before discharge, earlier than 1 month after surgery, with an intra-operative mortality of 15.5%, 105 patients were discharged with survival of 82, 60 and 54%, respectively, at 1, 2, and 3 years after implantation [the remaining 15 patients were either transplanted before discharge (5) or died in hospital (10)].

**Figure 9:**
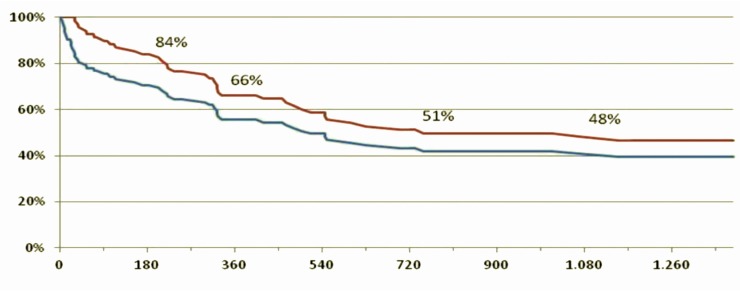
**Survival curves of patients included in the DAVID protocol, given the reported survival in the INTERMAS 2011 (45)**. Blue line = subjects who underwent implantation of the device (*n* = 143). Red line = survival of those discharged (*n* = 105).

Figure 9 shows the survival curve of patients with Jarvik 2000^®^ in the medium-long term, a similar survival curve to that reported by the INTERMACS 2014 study (45).

**Figure 10:**
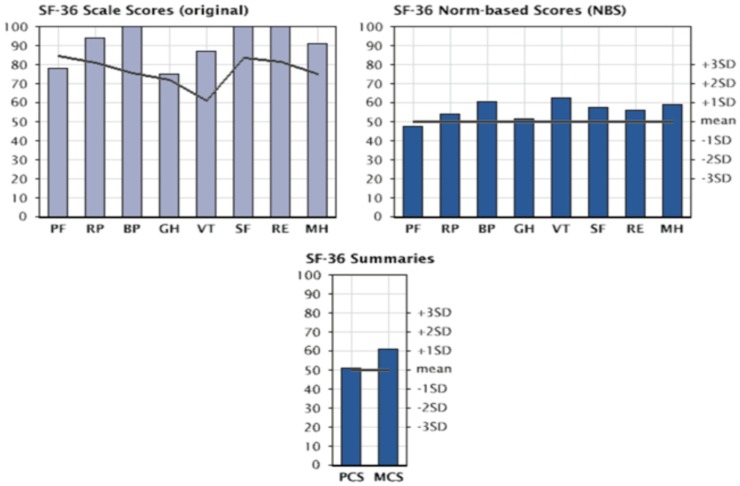
**Evaluation of quality of life in patients with Jarvik 2000 via SF-36 questionnaire: graphs are presented as following; SF-36 score (in upper left), SF-36 norm-based score (upper right), and SF-36 summaries (bottom) at 9 months from Implant of the Jarvik 2000^®^**. PF, physical functioning; RP, physical role; BP, bodily pain; GH, general health; VT, vitality; SF, social functioning; RE, emotional role; MH, mental health; PCS, physical health component summary; MCS, mental health component summary.

The assessment of quality of life (QoL) in patients with Jarvik 2000^®^ was carried out with the SF-36 questionnaire: a multidimensional survey divided into 36 questions that allows an in-depth analysis of the various physical and psychological components. The questionnaire was administered to patients at 3, 6, and 9 months after implantation of the Jarvik 2000^®^. Figure 10 shows the scores obtained at 9 months of implantation of the VAD for individual components, component summary *physical health component summary* (PCS) and *mental health component summary* (MCS). These values are normalized and compared with the values obtainable in a healthy population with the same characteristics of age and gender distribution.

The most obvious improvements concern the field of physical health, expressed as the ability to carry out activities without limitations due to the physical condition and with the absence of pain. These are seen between the third and the sixth month from implantation. At 9 months from implantation all parameters examined were showing improvements, thus validating VAD’s capability for real Bridge to Life.

**Figure 11:**
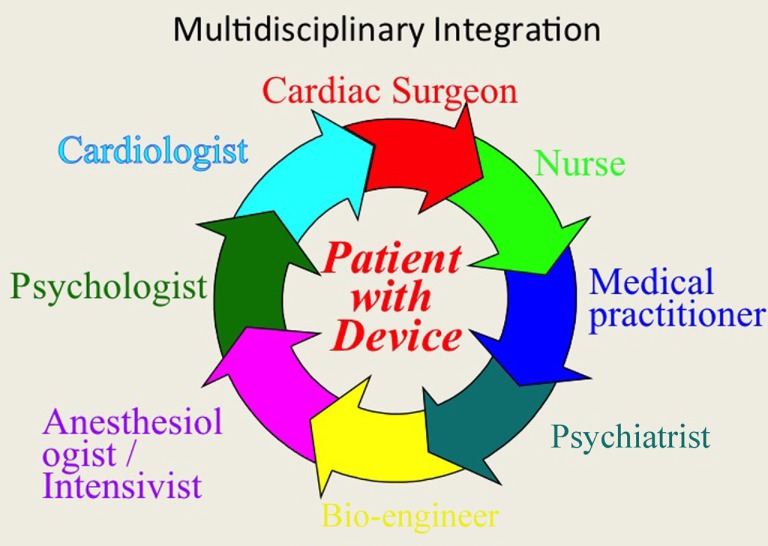
**The multidisciplinary integration, as well as the technological evolution of VAD will be essential in the coming years to compare treatment of mechanical assist devices to transplantation in terms of survival and quality of life**.

As we wait for the final results, it is necessary for us to optimize the clinical care strategy of patients who undergo VAD implantation. We stress the need for a multidisciplinary approach to the patient with end-stage heart failure, with close collaboration between various professionals in order to improve all phases of therapy (Figure 11): from design to production of more biocompatible devices, to pre-implant evaluation systems, from postoperative management to the reintegration of the patient into an active and productive life. This approach is probably the most important key factor toward full implementation and success for this challenging and fascinating new therapeutic frontier.

## Conflict of Interest Statement

The authors declare that the research was conducted in the absence of any commercial or financial relationships that could be construed as a potential conflict of interest.
